# Impact of SARS-CoV-2 Variant NSP6 on Pathogenicity: Genetic Analysis and Cell Biology

**DOI:** 10.3390/cimb47050361

**Published:** 2025-05-14

**Authors:** Yangye Gao, Peng Ni, Yanqiao Hua, Shuaiyin Chen, Rongguang Zhang

**Affiliations:** 1 Department of Epidemiology, College of Public Health, Zhengzhou University, Zhengzhou 450001, China; gyy20913@gs.zzu.edu.cn (Y.G.); nipeng17@163.com (P.N.); huayanqiaohyq@163.com (Y.H.); sychen@zzu.edu.cn (S.C.); 2 School of Public Health, Key Laboratory of Tropical Translational Medicine of Ministry of Education, Hainan Medical University, Haikou 570100, China

**Keywords:** SARS-CoV-2, nonstructural protein 6, gene mutation, bioinformatics, transcriptomics, cell signaling pathway

## Abstract

SARS-CoV-2 nonstructural protein (NSP) 6 is one of the factors affecting viral pathogenicity. Mutations in NSP6 continuously emerge during viral transmission and are closely associated with alterations in viral pathogenicity. This study investigated the structural and functional impacts of NSP6 mutations by analyzing NSP6 proteins from the Wuhan-Hu-1/B (WT) strain and predominant variants Alpha, XBB.1.16, BA.2.86, and JN.1 using bioinformatics, transcriptomics, and cellular experiments. The results demonstrate that the V3593F mutation decreased the β-sheet proportion and modified hydrogen bonding patterns, while the L3829F mutation enhanced structural stability by promoting random coils. The R3821K substitution exposed lysine residues, potentially enhancing molecular interactions. Combined transcriptomic profiling and functional assays revealed that WT-NSP6 significantly inhibited poly (I: C)-induced immune factor transcription and reduced the phosphorylation levels of p-IRF3 and p-STAT1, effects absent in the XBB.1.16 variant. Furthermore, WT-NSP6 markedly activated p-AKT and p-mTOR expression, with JN.1-NSP6 maintaining limited capacity to upregulate p-mTOR. However, p53 inhibitor treatment reversed Alpha-NSP6- and BA.2.86-NSP6-upregulated p-mTOR protein expression in cells. This study demonstrates that a high frequency of NSP6 mutations alters NSP6’s structure, impairing the type I interferon signaling pathway and affecting host antiviral responses through the p53-AKT-mTOR signaling pathway. These findings contribute to the understanding of evolution, immune evasion, and viral pathogenesis mechanisms, with potential implications for the development of antiviral therapies and preventive strategies for this viral infection.

## 1. Introduction

Following the initial identification of severe acute respiratory syndrome coronavirus 2 (SARS-CoV-2) in Wuhan, China, during late 2019, the public crisis and spread of COVID-19 outbreaks have had a profound impact on the entire human community. In 2024, the World Health Organization (WHO) issued a global outbreak emergency warning that the epidemic continued to spike during the Olympics. The report said that the fluctuation in COVID-19 showed an alternating six-month cycle, becoming a phased epidemic respiratory disease.

SARS-CoV-2, which belongs to the beta-coronavirus genus, is a positive-sense single-stranded RNA virus characterized by an enveloped structure, 100–160 nm particle diameter, and a genomic sequence spanning 27–32 kb [1]. The viral genome orchestrates the synthesis of 29 proteins, comprising 9 accessory proteins, 4 structural components, and 16 nonstructural proteins (NSP1-16). Positioned at nucleotides 266-21555, spanning the initial 67% of the genomic architecture, the ORF1ab reading frame governs the synthesis of nonstructural proteins (NSPs) critical for coronaviral RNA replication–transcription machinery assembly and transcriptional modulation [2].

NSPs serve as critical mediators of viral pathogenicity by compromising host cell integrity while simultaneously driving viral component biosynthesis and virion assembly [1]. Among them, NSP6, a membrane-spanning protein conserved across α- and β-coronavirus lineages, functions to facilitate viral replication compartment assembly through its structural integration into the viral factory architecture [3], with a total of 127 unstable mutations and 142 stable mutations distributed throughout the structure [4]. NSP6 emerges as a critical virulence modulator in SARS-CoV-2 pathogenesis [5], and is a central modulator of SARS-CoV-2 Omicron BA.1, which exhibits reduced virulence through virulence regulation. This transmembrane protein drives NLRP3-mediated pyroptotic cascades and proinflammatory cytokine release in pulmonary tissues of infected individuals, establishing its dual role as both an immunopathological instigator and essential virulence component in COVID-19 progression [6]. The literature suggests that NSP6 mutations may be correlated with adverse clinical trajectories in COVID-19 patients [7] and could also lead to considerable modifications in how SARS-CoV-2 expresses itself in its host, specifically involving key host antiviral defenses [3]. The deletion and mutation of NSP6 may be to adapt to replication in humans, on the one hand, to avoid elimination by the host immune system, or, on the other hand, to hijack the host protein expression system for replication and packaging [8]. The L37F mutation, notably associated with mild or asymptomatic infections, exerts its effects by disrupting interferon (IFN) signaling pathways and compromising the secondary structural stability of NSP6 [4,9]. Furthermore, this mutation diminishes NSP6’s ability to induce NLRP3-dependent pyroptosis while weakening cellular defenses through autophagy modulation, collectively reducing viral pathogenicity [10].

In this study, bioinformatics technology was applied to compare and analyze the transmembrane structural domains, protein chemical remodeling, protein structure, and stability of WT-NSP6 and JN.1-NSP6 (V3593F + ΔSGF + R3821K), BA.2.86-NSP6 (V3593F + ΔSGF), and XBB.1.16-NSP6 (L3829F + ΔSGF) to evaluate how mutations in the nsp6 gene affect protein activity. Plasmids carrying the nsp6 genes of different SARS-CoV-2 variants were constructed and transfected into human non-small cell lung cancer cells (A549 cells). Combined with transcriptome sequencing analysis, the diversity in the nsp6 genes of SARS-CoV-2 variants and their effects on cell signaling pathways were investigated. The objective of this study is to determine the role of the nsp6 gene in SARS-CoV-2 pathogenicity and evaluate the effects of its genetic variations on viral virulence, thereby providing critical evidence for epidemic prevention and control.

## 2. Materials and Methods

### 2.1. Comparative Analysis of Predominant Variant Sequences and Compilation of nsp6 Gene Mutant Sites

The classifications of variants of concern (VOCs), variants of interest (VOIs), and variants under monitoring (VUMs) as of December 2024 were obtained and compiled from WHO COVID-19 reports. Comprehensive genetic sequences of predominant SARS-CoV-2 variants across the globe as of 1 December 2024 were downloaded from the following databases: the SARS-CoV-2 Data Hub of the National Center for Biotechnology Information (NCBI, Bethesda, MD, USA), the Global Initiative of Sharing All Influenza Data (GISAID, Munich, Germany), and the 2019 Novel Coronavirus Information Database of the National Genomics Data Center (NGDC, Boulder, CO, USA). A total of 2,366,045 sequences were collected to establish the corresponding database. The Outbreak.info online tool (https://outbreak.info/, accessed on 1 December 2024) was employed to visualize the mutation frequencies of dominant variants as a heatmap. Mutations with a frequency > 75% were defined as dominant mutation sites, and the earliest reported variant carrying each mutation was selected as the representative strain [11]. Genomic and protein sequence alignment and comparative analyses were performed using Clustal W 2.1 software, with the SARS-CoV-2 Wuhan-Hu-1 strain (NC_045512, NCBI) as the reference sequence. The comparison results were visualized using Genedoc software (version 2.7) to obtain gene and protein mutation site changes.

### 2.2. Phylogenetic Tree Construction

MEGA11 (Molecular Evolutionary Genetics Analysis, New York, NY, USA) software (version 11.0.13) is a widely used bioinformatics tool, which is mainly used for multiple sequence comparisons, molecular evolution, and phylogenetic analyses [12]. The sequence files in FASTA format were imported into this software for phylogenetic tree construction. The Maximum Likelihood (ML) method was employed for tree construction, with model selection conducted through the “Find Best DNA/Protein Models (ML)” module. The optimal substitution model, determined by the Bayesian Information Criterion (BIC), was identified as “Tamura-Nei 93 (TN93) + Gamma-distributed rate variation across sites (G, 4 rate categories) + proportion of invariant sites (I)”. Tree topology optimization utilized the Nearest-Neighbor Interchange (NNI) search algorithm, with branch support values evaluated through 1000 bootstrap replicates.

### 2.3. Bioinformatics Analysis

The characteristics of the NSP6 protein in SARS-CoV-2 variants, including transmembrane helices, spatial structure, protein stability, pathogenicity, flexibility, and antigenic epitopes, were predicted using online bioinformatics tools. Comprehensive details are presented in Table 1.

### 2.4. Plasmid Construction

The plasmids pcDNA3.1-WT-NSP6 (NCBI accession: NC_045512), pcDNA3.1-Alpha-NSP6 (NCBI accession: MW422255.1), pcDNA3.1-XBB.1.16-NSP6 (NCBI accession: BS007330.1), pcDNA3.1-BA.2.86-NSP6 (NCBI accession: OR537351.1), and pcDNA3.1-JN.1-NSP6 (NCBI accession: OR708362.1) were downloaded from the NCBI database and synthesized by Sangon Biotech (Shanghai, China); the empty vector, pcDNA3.1, was preserved by the research group.

### 2.5. Cell Culture

A549 cells were preserved in the laboratory. A549 cells were cultured in complete medium consisting of DMEM medium (Gibco, Grand Island, NY, USA), 10% fetal bovine serum (BioChannel, Nanjing, China), and 1% penicillin–streptomycin (Pricella, Wuhan, China). Cells were cultured at 37 °C with 5% CO_2_ for 2–3 days until they reached 90% confluence. The supernatant in the culture dish was discarded and washed twice with 2 mL of sterile PBS. Then, 750 μL of 0.25% trypsin (Solarbio, Beijing, China) was added and digested for 3 min in an incubator, followed by the addition of 1 mL of complete medium to terminate the digestion. Blow the cells gently to dislodge them and transfer them into a freezing tube, centrifuge at 1000 rpm for 5 min, discard the supernatant, and resuspend the cells by adding 1 mL of complete medium and passaging according to 1:2 or 1:3 as required.

### 2.6. Cell Transfection

A549 cells in the logarithmic growth phase with optimal viability were selected for transfection. After trypsinization and centrifugation, cells were counted using trypan blue solution (Solarbio, Beijing, China). Cells were inoculated into 6-well plates at a density of 8 × 10^5^ cells/well with 2 mL complete medium. After the cell density reached 70–80%, the plasmid was transfected into the A549 cells, and the complete culture medium was replaced with cell culture medium after 6 h. Cells were harvested 48 h after transfection, and successful overexpression of NSP6 was confirmed by Western blotting using an anti-His tag antibody.

### 2.7. Transcriptome Sequencing Analysis

The plasmid was re-transfected in the A549 cells, and after 24 h, the cells were lysed using RNAiso Plus (Takara Bio, Kusatsu, Japan) and collected in 1.5 mL sterile RNase-free microcentrifuge tubes. Samples were flash-frozen in liquid nitrogen, stored at −80 °C, and subsequently sent to Novogene Co., Ltd. (Beijing, China) for transcriptome sequencing. Differential gene expression analysis between groups was performed using DESeq2 (version 1.20.0), with a significance threshold of |log_2_(fold change)| ≥ 0.58 and adjusted *p*-value ≤ 0.05 after Benjamini–Hochberg correction for the false discovery rate (FDR). Gene Ontology (GO) enrichment analysis of differentially expressed genes (DEGs) was conducted with clusterProfiler (version 3.8.1), incorporating gene length bias correction. Pathway enrichment analysis for KEGG (Kyoto Encyclopedia of Genes and Genomes) was similarly performed using clusterProfiler (version 3.8.1).

### 2.8. Cell Stimulation and Inhibition Assays

Stock solutions of experimental reagents were prepared as follows: the interferon inducer Poly(I:C) (HY-107202, MedChemExpress, Monmouth Junction, NJ, USA) was diluted to 1 g/L using sterile ultrapure water, with a working concentration of 500 ng/mL; the JAK/STAT pathway agonist RO8191 (HY-W063968, MedChemExpress) was dissolved in DMSO (HY-Y0320C, MedChemExpress) to 10 mM stock solution and further diluted to 10 μM working concentration (final DMSO concentration ≤ 0.1%); the p53 inhibitor Pifithrin-α (S1816, Biotechnology, Shanghai, China) was prepared in DMSO as a 20 mM stock solution and diluted to 20 μM working concentration.

A549 cells were taken after 24 h of plasmid transfection, and when the cell confluence reached 80%, the culture medium was replaced with fresh medium containing agonists or inhibitors under sterile conditions. Poly (I:C)-treated cells were incubated for 10 h at 37 °C with 5% CO_2_, while the RO8191- and Pifithrin-α-treated groups were incubated for 24 h. Following treatment, cells were harvested for RNA extraction to quantify cytokine mRNA levels and for protein isolation to assess key signaling pathway protein expression via qRT-PCR and Western blotting, respectively.

### 2.9. Western Blotting

Cultured cells in optimal condition were removed from the incubator, and the spent medium was aspirated. Cells were washed 2–3 times with 1–2 mL of pre-cooled sterile PBS per well. Then, 100 μL of pre-cooled SDS lysate with protease inhibitor (Biotechnology, Shanghai, China) was added to each well and lysed on ice for 10~15 min. The sample was collected into 1.5 mL sterile RNase-free microcentrifuge tubes. Afterwards, 2× protein sample buffer (Solarbio, Beijing, China) was added and boiled for 15 min in a metal bath, followed by a brief period of centrifugation. The prepared protein samples were separated by gel electrophoresis using the SDS-PAGE gel preparation kit (Zhonghui Hecai Biomedical Technology Co., Ltd., Xi’an, China) and transferred to a 0.22 μm nitrocellulose membrane. Membranes were blocked with 5% skim milk (prepared in distilled water) at room temperature for 90 min. The membranes were incubated overnight at 4 °C or incubated for 3 h at room temperature using the following specific primary antibodies: His tag (1:1000, 1B7G5, Proteintech, Wuhan, China), RIG1 (1:1000, D14G6, Cell Signaling Technology, Boston, MA, USA), MDA5 (1:1000 D74E4, Cell Signaling Technology), TBK1 (1:1000, D1B4, Cell Signaling Technology, Boston, USA), p-TBK1 (1:1000, D53C2, Cell Signaling Technology), AKT (1:1000, C67E7, Cell Signaling Technology), p-AKT (1:1000, D9E, Cell Signaling Technology), mTOR (1:1000, 7C10, Cell Signaling Technology), p-mTOR antibody (1:1000, D9C3, Cell Signaling Technology, Boston, USA), IRF3 (1:500, 381333, ZEN-BIOSCIENCE, Chengdu, China), p-IRF3 (1:500, R381561, ZEN-BIOSCIENCE, Chengdu, China), p-STAT1 (1:500, 340797, ZEN-BIOSCIENCE, Chengdu, China), GAPDH (1:1000, ab8245, Abcam, Cambridge, UK). Membranes were washed three times with TBST (10 min per wash) and then incubated with horseradish peroxidase (HRP)-conjugated secondary antibody (1:5000, Zen-Bioscience, Chengdu, China) at room temperature for 1 h. After three additional TBST washes (10 min each), membranes were incubated with ECL working solution (Biosharp, Beijing, China) for 1–2 min. Protein bands were visualized using a chemiluminescence imaging system and quantified with ImageJ software (version 1.53a, National Institutes of Health, Bethesda, MD, USA).

### 2.10. Quantitative Real-Time PCR, qRT-PCR

Total RNA was extracted from cells using TRIzol reagent. RNA concentration and purity were assessed by UV spectrophotometry (A260/A280 ratio ≥ 1.8). Reverse transcription was performed using the HiScript III RT SuperMix kit (Vazyme, Nanjing, China) according to the manufacturer’s protocol. Cytokine mRNA expression levels were quantified using the SYBR qPCR Master Mix kit (Vazyme, Nanjing, China), with GAPDH as the endogenous control. Primer sequences for target cytokines are listed in Supplementary Table S1. Amplification curves were analyzed to determine the relative expression levels of target genes normalized to GAPDH. The cycle threshold (Ct) values were calculated from amplification curves, and relative gene expression was determined using the 2^−ΔΔCt^ method.

### 2.11. Statistical Analysis

SPSS 21.0 software was utilized for statistical analysis, and GraphPad Prism 9.0 software was used for data visualization. Normality-assessed datasets with multigroup comparisons underwent one-way ANOVA, supplemented by Fisher’s LSD post hoc tests for pairwise comparisons under significance criteria (α = 0.05).

## 3. Results

### 3.1. Assessment of Predominant SARS-CoV-2 Variant Sequences and Summary of NSP6 Mutation Locations

**Figure 1:**
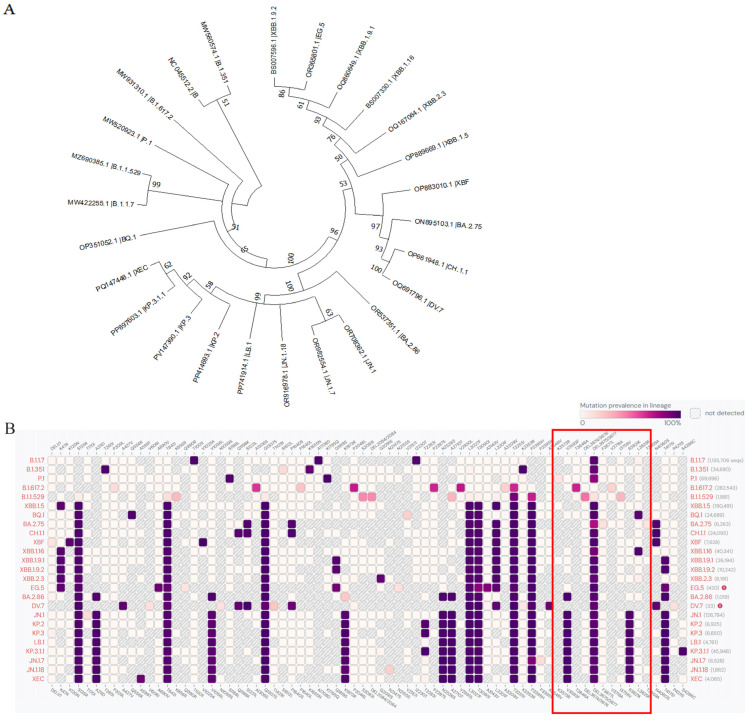
(**A**) Phylogenetic tree of the 25 dominant variants. (**B**) Mutation frequency of sites in the ORF1a region in the 25 variants, with the NSP6 mutation regions highlighted in red boxes. The intensity of purple indicates the mutation frequency for each variant.

Since the emergence of the COVID-19 pandemic, the World Health Organization (WHO) has classified Alpha (B.1.1.7), Beta (B.1.351), Gamma (P.1), Delta (B.1.617.2), Omicron (B.1.1.529), and their subvariants as variants of concern (VOCs). For variants emerging after 15 March 2023, the WHO categorizes them into variants of interest (VOIs) and variants under monitoring (VUMs) based on changes in virulence, clinical disease manifestations, or public health impact. As of December 2024, a total of 25 predominant strains have been obtained (Table S2). A phylogenetic tree was constructed using MEGA software (version 11.0.13) (Figure 1A). Phylogenetic analysis revealed that BA.2.86/JN.1 exhibited a greater genetic distance from the wild-type (WT) strain compared to XBB.1.16, with BA.2.86/JN.1 also displaying a longer genetic divergence from XBB.1.16. The comparative results of the collected predominant variant gene sequences (amino acid mutations with >5% frequency) are visualized as a heatmap, with NSP6 mutant sites annotated (Figure 1B).

**Figure 2:**
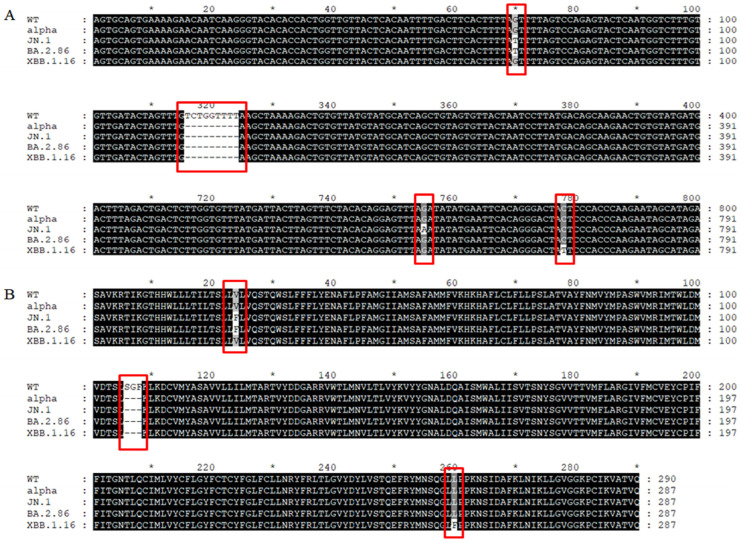
(**A**) Comparative analysis of nucleotide sequences of NSP6 in the studied variants, using the WT-NSP6 sequence as the reference, with mutant sites highlighted in red boxes. (**B**) Comparative analysis of amino acid sequences of NSP6 in the studied variants, using the WT-NSP6 sequence as the reference, with mutant sites highlighted in red boxes. “*” was used to mark the position of every tenth site in the sequence.

The selected predominant variants and their NSP6 mutant sites for analysis include JN.1 (V3593F + ΔSGF + R3821K), BA.2.86 (V3593F + ΔSGF), and XBB.1.16 (L3829F + ΔSGF), using Wuhan-Hu-1/B (WT) and Alpha (ΔSGF) as references. ClustalW was used to align the NSP6 nucleotide and amino acid sequences of WT, Alpha, XBB.1.16, BA.2.86, and JN.1, with visualization performed using Genedoc (Figure 2). Compared to Wu-han-Hu-1/B (WT), V3593F was g.11042G>T (valine-to-phenylalanine substitution), R3821K was g.11727G>A (arginine-to-lysine substitution), L3829F was g.11750C>T (leucine-to-phenylalanine substitution), and ΔSGF was g.11288–11296del (designated as NSP6Δ106-108, p.3675–3677del, resulting in the deletion of serine, glycine, and phenyl-alanine).

### 3.2. Transmembrane Domain Prediction Analysis

**Figure 3:**
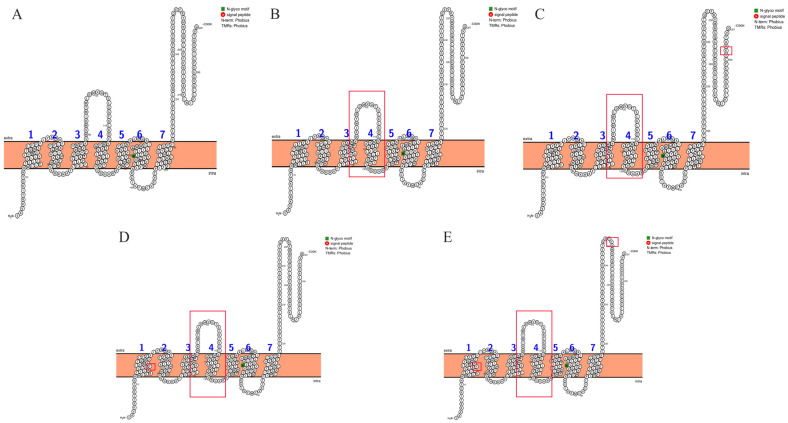
Predicted topology of NSP6 proteins in different variant strains. Panels (**A**–**E**) correspond to the WT, Alpha, XBB.1.16, BA.2.86, and JN.1 strains, respectively. The location of the mutation site is shown in the red box. Numbers 1–7 indicate the number and location of protein transmembrane.

TMHMM2.0 was employed to determine the transmembrane regions of NSP6 mutant proteins, and the protein topology was predicted using the Protter server (https://wlab.ethz.ch/protter/#, accessed on 22 August 2024). The results showed that NSP6 proteins of different variants contained transmembrane regions with seven transmembrane helices, and belonged to transmembrane proteins (Figure 3).

### 3.3. Protein Structure Prediction

**Figure 4:**
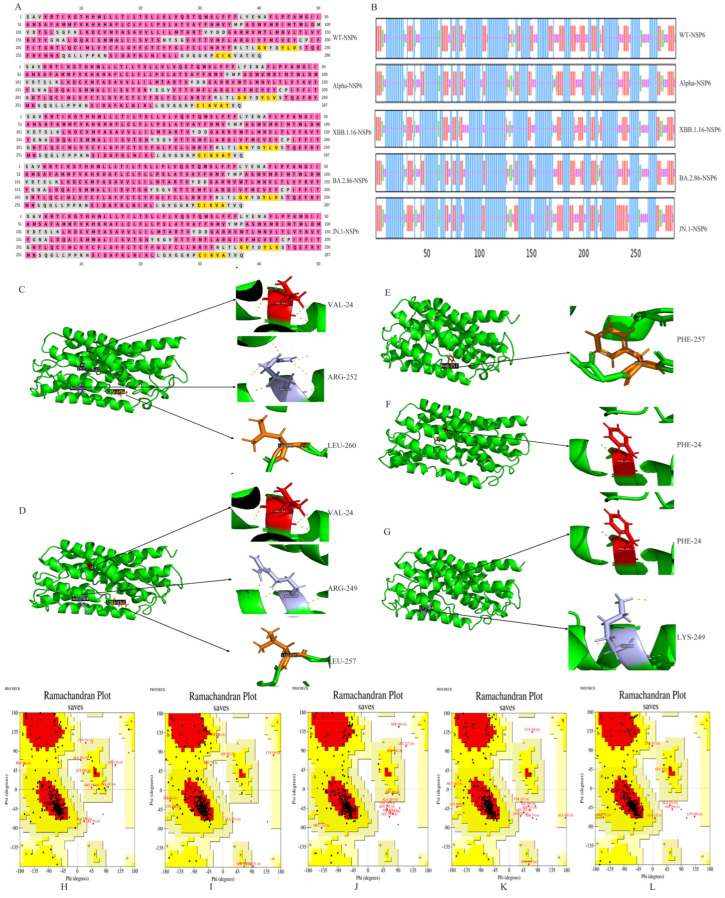
NSP6 protein structure prediction results for SARS-CoV-2 variants. (**A**) shows the PRISED-predicted secondary structures of NSP6 proteins from five variants. The colors are as follows: pink for alpha helix, gray for random coil, and yellow for extended strand. (**B**) displays the SOPMA-predicted secondary structures of NSP6 proteins from five variants. The colors are as follows: blue represents alpha helix, green corresponds to beta sheet, yellow indicates random coil, and red indicates extended strand. (**C**–**G**) display the tertiary structure predictions and stick model visualizations of mutant sites. The arrow indicates the enlarged view of the mutation site, where the dashed lines in the diagram represent hydrogen bonds. (**H**–**L**) present Ramachandran plot evaluations for the tertiary structures.

The secondary structures of NSP6 proteins from different variants were predicted using the online software PSIPRED (http://bioinf.cs.ucl.ac.uk/psipred/, accessed on 27 August 2024) and SOPMA (https://npsa-prabi.ibcp.fr/cgi-bin/npsa_automat.pl?page=npsa_sopma.html, accessed on 26 August 2024) (Figure 4A–B). The results revealed that the secondary structure of WT-NSP6 contains 50.69% α-helices, 26.21% extended chains, 5.17% β-folding, and 17.93% random coils. All four mutant sites in NSP6 altered the secondary structure, increasing the α-helix content while reducing extended chains. The V3593F mutation site decreased the β-folding content, and the L3829F mutation site increased the random coil proportion (Table S3).

Tertiary structure models of NSP6 were forecasted using the I-TASSER server and validated using SAVES (Figure 4H–L). The Ramachandran plot showed that over 90% of the amino acid residues fell within the most favored and allowed regions, with less than 5% in disallowed regions, confirming high model accuracy.

PyMOL (3.0.4) was used to visualize the 3D structural models of the proteins before and after mutations (Figure 4C–J), with mutant sites displayed as stick models. The V3593F, R3821K, and L3829F mutant sites caused significant shifts in the proteins’ carbon skeleton. Structural changes were particularly notable for V3593F and L3829F, with V3593F in particular causing large changes to the hydrogen bonds formed. This suggests that the mutant sites may affect the structure or other properties of the proteins.

### 3.4. Prediction of Protein Structural Pathogenicity, Stability, and Flexibility

Missense3D was applied to predict harmful structural alterations caused by amino acid variations (Table 2). The mutations were predicted to induce three potentially deleterious structural alterations. “Buried H-bond breakage” indicates that the V3593F mutation disrupts all backbone hydrogen bonds originally formed by the buried VAL residues (RSA 3.5%). “Buried/exposed switch” shows that the R3821K mutation causes the target residue to switch from a buried state (ARG, RSA 2.4%) to an exposed state (LYS, RSA 14.6%). “Buried/exposed switch” indicates that the R3821K mutation site induces a transition of the target residue from a buried state (ARG, RSA 2.4%) to an exposed state (LYS, RSA 14.6%) “Cavity altered” reveals that the V3593F mutation reduces cavity volume by 81.864 Å^3^.

Polyphen-2 predicted that R3821K and L3829F are highly likely to alter protein structure and function, while SIFT analysis indicated that V3593F and R3821K significantly disrupt protein function compared to WT-NSP6, and R3821K and L3829F showed greater disruptive effects relative to Alpha-NSP6 (Table 3).

The Mupro (https://mupro.proteomics.ics.uci.edu/, accessed on 10 September 2024) and SAAFEC (http://compbio.clemson.edu/SAAFEC-SEQ/, accessed on 17 September 2024) tools predicted negative free energy changes (ΔΔG < 0) for all three mutant sites, suggesting reduced stability of NSP6. The DUET server was employed to calculate free energy changes induced by mutations, predicting that all three examined NSP6 variants (relative to WT-NSP6 and Alpha-NSP6) would decrease protein stability (ΔΔG < 0) and compromise functional integrity. TM-Align calculated root mean square deviation (RMSD) values > 0.15 for all mutations, indicating potential impacts on protein functionality (Table 4). PredyFlexy predicted increased B-factor values at the R3821K and V3593F mutant sites, implying elevated local flexibility and conformational instability (Table 5).

### 3.5. Protein Antigenicity Analysis

**Figure 5:**
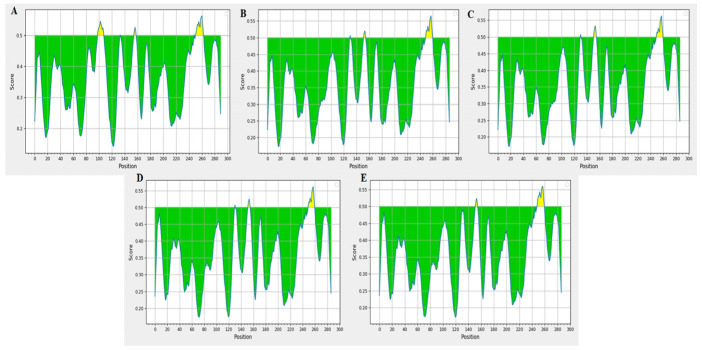
Predicted B-cell epitopes of NSP6 proteins of five SARS-CoV-2 variants. (**A**–**E**) correspond to WT, Alpha, XBB.1.16, BA.2.86, and JN.1 strains, respectively. On the graphs, the Y-axes depicts for each residue the correspondent score, while the X-axes depicts the residue positions in the sequence. The larger score for the residues might be interpreted as that the residue might have a higher probability to be part of epitope (those residues are colored in yellow on the graphs).

B-cell epitopes of NSP6 proteins from variants were predicted using IEDB (Figure 5). The results identified five B-cell antigenic epitopes for WT-NSP6, three for Alpha-NSP6, three for XBB.1.16-NSP6, three for BA.2.86-NSP6, and two for JN.1-NSP6. The variation in epitope numbers may correlate with pathogenicity. Cytotoxic T lymphocyte (CTL) epitopes were predicted using IEDB and SYFPEITHI, with results filtered for IEDB scores ≥ 0.5 and SYFPEITHI scores ≥ 25 (Table S4). All variants of NSP6 proteins retained eight antigenic sites, though changes in epitope scores due to the ΔSGF deletion may be related to pathogenicity.

### 3.6. Identification of pcDNA3.1-NSP6-His Plasmids and Protein Expression

**Figure 6:**
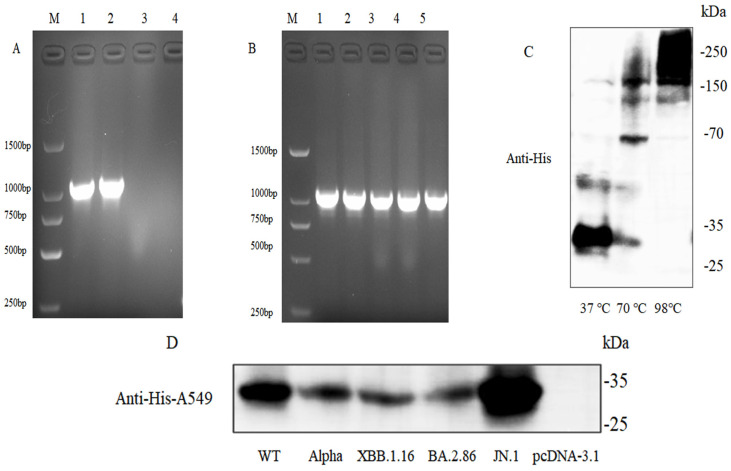
Identification and protein expression of A549 cells transfected with NSP6 plasmid. (**A**) Gel electrophoresis of NSP6 plasmid PCR products. M: marker; lanes 1, 2: WT-NSP6; lane 3: pcDNA3.1; lane 4: negative control. (**B**) Gel electrophoresis of PCR products from NSP6 plasmids of different variants. M: marker; lanes 1–5: WT–NSP6, Alpha–NSP6, XBB.1.16–NSP6, BA.2.86–NSP6, JN.1–NSP6. (**C**) NSP6 protein expression under different temperature conditions. (**D**) Protein expression of different variants of NSP6-transfected A549 cells.

The codon-optimized nsp6 gene sequences of different variants were cloned into the eukaryotic expression vector pcDNA3.1, with a His-tag fused to the C-terminus of each gene. PCR validation (Figure 6A,B) confirmed successful cloning, showing approximately 1000 bp bands for all five plasmids, consistent with expected sizes. The target proteins were harvested by transfection of A549 cells and lysed for Western blot analysis, which detected pcDNA-NSP6-his expression at the expected molecular weight in unboiled samples, while no expression was observed at the expected size in boiled samples (Figure 6C). Boiling likely denatured NSP6 proteins, causing aggregation mediated by hydrophobic transmembrane domains (TMs) and increased molecular weight. Expression of NSP6 plasmids carrying other variants in transfected A549 cells was also confirmed (Figure 6D).

### 3.7. Transcriptome Sequencing Analysis

**Figure 7:**
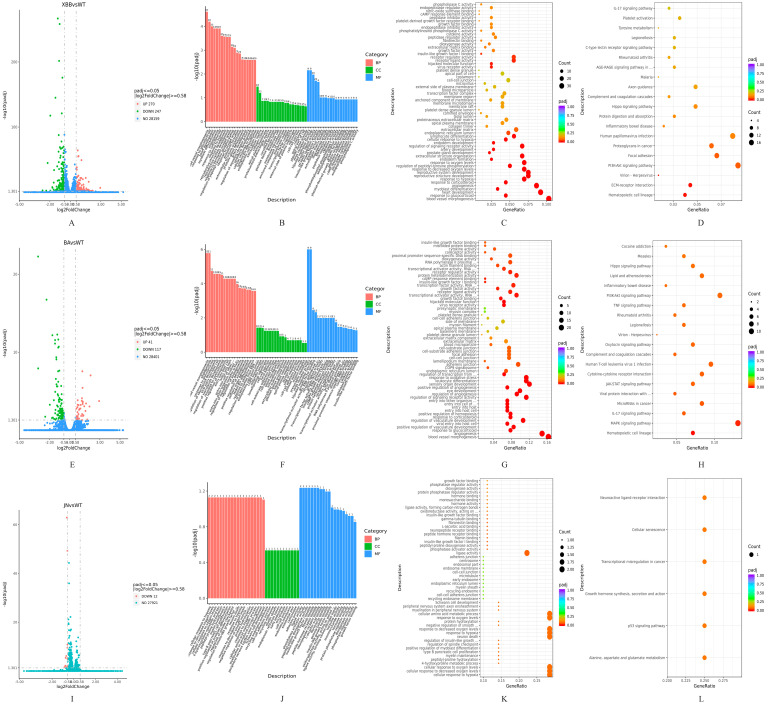
Transcriptomic analysis of NSP6 variants. (**A**,**E**,**I**) Volcano plots comparing XBB.1.16–NSP6, BA.2.86–NSP6, and JN.1–NSP6 to WT–NSP6, respectively. The dashed lines indicate the threshold of |log_2_(fold change)| > 0.58. (**B**,**F**,**J**) GO enrichment bar charts. (**C**,**G**,**K**) GO enrichment scatter plots. (**D**,**H**,**L**) KEGG enrichment scatter plots.

Transcriptome sequencing was performed after transfection of A549 cells with WT-NSP6 and different variants of NSP6 plasmids. Differentially expressed genes (DEGs) were identified applying adjusted *p* < 0.05 and |log_2_ (fold change)| > 0.58. The volcano plots (Figure 7A,E,I) revealed 517 differential genes in the XBB.1.16-NSP6 group (270 upregulated, 247 downregulated), 158 differential genes in the BA.2.86-NSP6 group (41 upregulated, 117 downregulated), and 12 differential genes in the JN.1-NSP6 group (all downregulated) compared to WT-NSP6. The differential genes were analyzed using qRT-PCR to detect the expression of the differential genes (Figure S1).

Functional annotation through Gene Ontology (GO) categorization and systems biology profiling via Kyoto Encyclopedia of Genes and Genomes (KEGG) pathway topology mapping were systematically employed for computational enrichment mapping of biological processes and molecular functions. The differential genes in XBB.1.16-NSP6 were enriched in biological processes (BPs) such as vascular morphogenesis, response to glucocorticoids, and response to corticosteroids (Figure 7B,C), and KEGG pathways including PI3K-AKT signaling, ECM–receptor interaction, and herpes simplex virus infection (Figure 7D). The differential genes in BA.2.86-NSP6 showed enrichment in molecular functions (MFs) like viral receptor activity and BP terms such as viral entry into host cells (Figure 7F,G), with KEGG pathways involving MAPK signaling, PI3K-AKT signaling, and JAK-STAT signaling (Figure 7H). The differential genes in JN.1-NSP6 were enriched in BP terms including neuronal action potential, myeloid leukocyte differentiation, and antimicrobial humoral response (Figure 7J,K), and KEGG pathways such as p53 signaling, transcriptional dysregulation in cancer, and cellular senescence (Figure 7L). These findings indicate divergent impacts of variant NSP6 proteins on cellular pathways, particularly in viral infection-related inflammation, immune responses, and the apoptosis signaling pathway.

### 3.8. Differential Suppression of Type I Interferon Signaling by Different Variants of NSP6

After transfection of A549 cells with either the pcDNA3.1 empty vector or plasmids carrying nsp6 genes from different variants, the cells were cultured for 24 h, followed by stimulation with poly(I:C) for 10 h. Total RNA was extracted to measure mRNA levels of interferons (IFNα, IFNβ), interferon-stimulated genes (ISG15, ISG56), interferon regulatory factors (IRF9), and chemokines (CXCL10). Protein extracts were analyzed for the expression and phosphorylation status of pattern recognition receptors (RIG-I, MDA5), signaling kinases (TBK1), and transcription factors (IRF3, STAT1).

**Figure 8:**
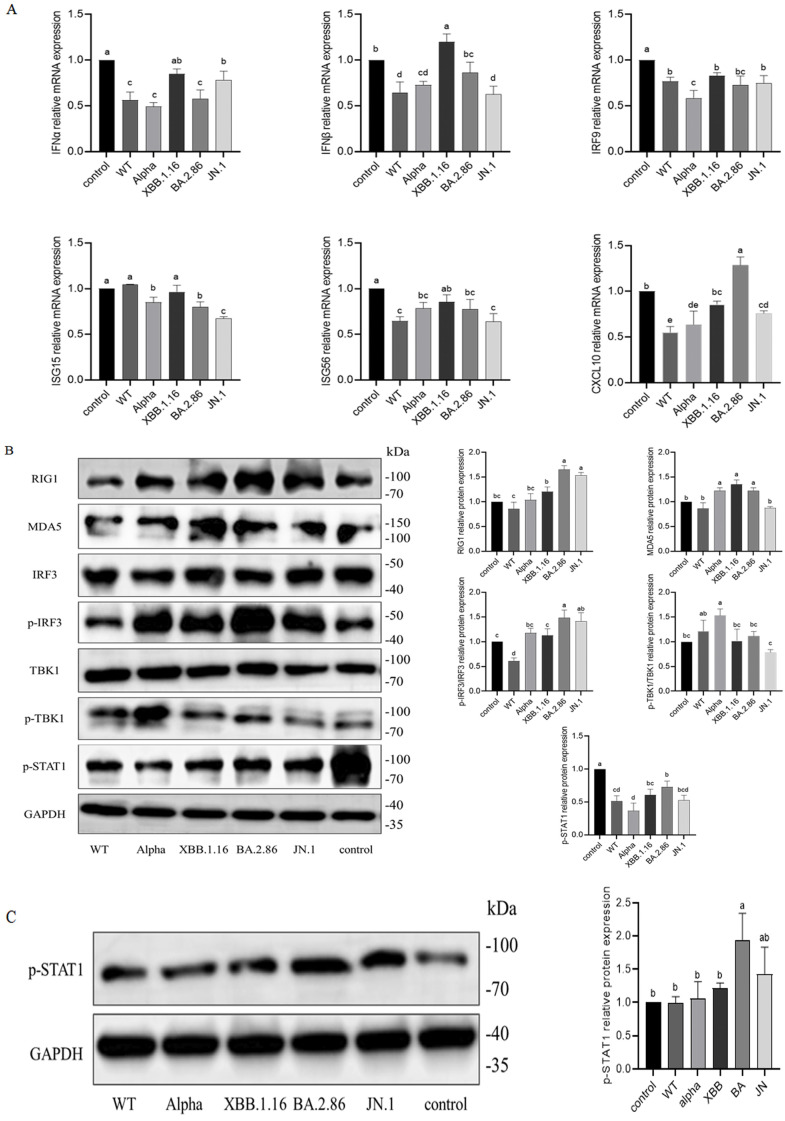
Effect of different variants of NSP6 on type I interferon signaling pathway in A549 cells. (**A**) Effects of NSP6 variants on mRNA expression of antiviral factors. (**B**) Impact of NSP6 variants on type I interferon signaling proteins. (**C**) p-STAT1 expression after RO8191 treatment. Groups sharing a common letter show no significant variation (*p* > 0.05); distinct letters denote significant differences (*p* < 0.05).

The results showed that plasmids encoding WT-NSP6, Alpha-NSP6, BA.2.86-NSP6, and JN.1-NSP6 significantly suppressed antiviral factor production (*p* < 0.05), while XBB.1.16-NSP6 attenuated this inhibition (*p* > 0.05) (Figure 8A). WT-NSP6 transfection markedly reduced p-IRF3 and p-STAT1 protein levels (*p* < 0.05), whereas other variants only suppressed p-STAT1 expression, with Alpha-NSP6 showing the strongest inhibition (Figure 8B). To further investigate the impact on the JAK-STAT pathway, cells were incubated with 10 μM RO8191 for 24 h post-stimulation. Western blot analysis revealed weakened suppression of p-STAT1 by NSP6 variants (Figure 8C).

### 3.9. Impact of NSP6 Variants on p53-AKT-mTOR Pathway in A549 Cells

**Figure 9:**
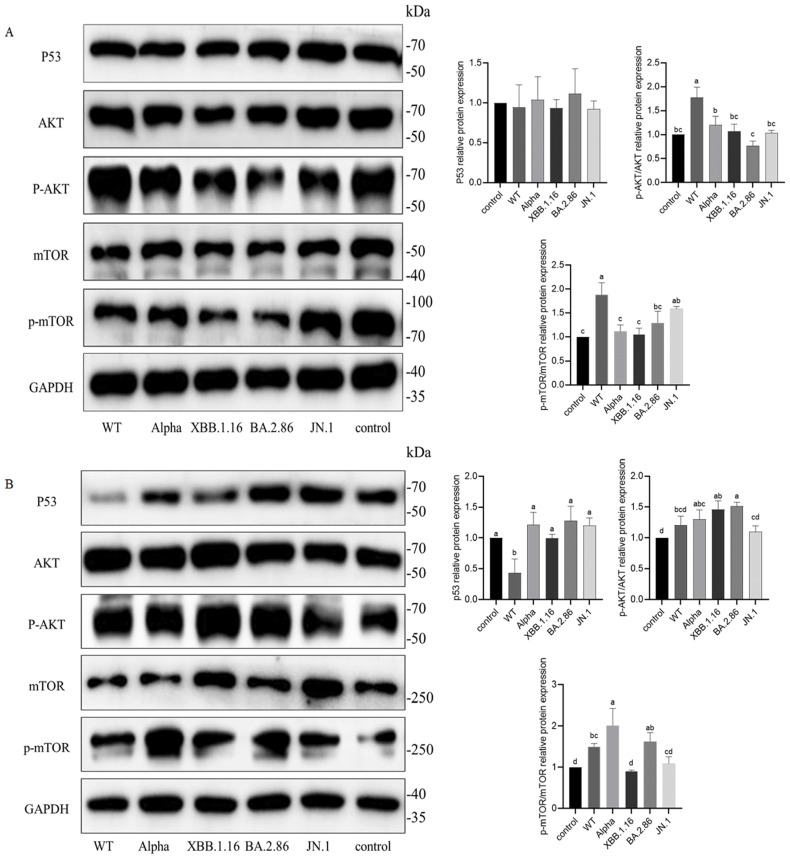
Effect of different variants of NSP6 on p53-AKT-mTOR pathway protein expression in A549 cells. (**A**) Protein expression in untreated A549 cells. (**B**) Protein expression in Pifithrin-α-treated A549 cells. Groups sharing a common letter show no significant variation (*p* > 0.05); distinct letters denote significant differences (*p* < 0.05).

To explore the impact of NSP6 on the p53-AKT-mTOR pathway, A549 cells were transfected with either the pcDNA3.1 empty vector or plasmids containing NSP6 genes from different variants. At 48 h post-transfection, proteins were collected and analyzed via Western blot. Compared to controls, transfection with WT-NSP6 plasmids significantly increased p-AKT and p-mTOR protein levels (*p* < 0.05), while JN.1-NSP6 plasmids elevated p-mTOR expression (*p* < 0.05) (Figure 9A). To further investigate the role of p53 in the pathway, cells were treated with 20 μM p53 inhibitor Pifithrin-α for 24 h after transfection. In cells transfected with Alpha-NSP6 or BA.2.86-NSP6 plasmids, p-mTOR levels were elevated significantly (*p* < 0.05) (Figure 9B).

## 4. Discussion

As an RNA virus, SARS-CoV-2 has a high rate of mutation at particular protein sites, leading to the rise of variants that intensify global health impacts [31]. Compared to early strains, SARS-CoV-2 variants exhibit extensive genomic mutations. Recombinant variants, like XBB and its derivatives, arise through co-infection of a host by distinct lineages, followed by genetic recombination [32]. XBB, which originated from BA.2.10.1 and BA.2.75, shows stronger immune evasion capabilities but has similar pathogenicity to earlier Omicron subvariants [33]. Since September 2023, dominant global strains have shifted from the XBB lineage to BA.2.86 [34], a descendant of BA.2 [33] representing a phylogenetically distinct lineage. JN.1 (BA.2.86.1.1) [35] replicates rapidly and efficiently in primary nasal epithelial cells, likely contributing to its selective advantage and heightened transmissibility [36]. It has been found that JN.1 demonstrates greater immune evasion than lineages derived from XBB [34].

Bioinformatics, a discipline integrating biological data with computer science for analysis [37], was used to evaluate the effects of amino acid mutations in NSP6 of SARS-CoV-2 on protein function, providing insights into the pathogenic differences among variants of NSP6 [38].

Nonstructural protein 6 (NSP6) is a transmembrane protein conserved in both α- and β-coronaviruses [2]. As a core functional component of biological membranes, its transmembrane architecture spans the phospholipid bilayer, playing pivotal roles in cellular energy transduction, material transport, and signal transduction [39]. Predictive analysis of transmembrane topology in SARS-CoV-2 variant NSP6 proteins not only elucidates their biological functions and evolutionary trajectories but also provides critical structural insights for addressing challenges posed by viral mutations. As a seven-pass transmembrane protein, NSP6 anchors via transmembrane helices to host cellular membranes or virus-induced membranous structures, directly participating in replication complex assembly and immune evasion mechanisms. Transmembrane topology prediction reveals the spatial localization of the mutation site and provides a basis for inferring its functional impact [40]. Mutations in the transmembrane region may affect membrane anchoring efficiency or membrane stability, whereas mutations at the extracellular end may regulate the protein’s interfaces with other viral components (e.g., NSP3/4) or host factors for interactions, thereby enhancing viral adaptability. Notably, all variants retained the conserved seven-pass transmembrane architecture, underscoring its indispensability for maintaining core functionality. This evolutionary conservation offers a theoretical foundation for designing broad-spectrum antiviral therapeutics targeting transmembrane domains or intervention strategies.

Missense mutations alter the primary amino acid sequence of proteins, which in turn affects the higher structure. Changes in the secondary structure of proteins affect stability and translational regulation, ultimately altering biological functions [41]. Additionally, changes in the secondary structure of proteins influence antigenic epitope formation, as random coils—often surface-exposed and structurally flexible—are more likely to interact with antibodies [42]. The tertiary structure of proteins, the three-dimensional conformation derived from the secondary structure of proteins, was predicted using I-TASSER, the most accurate homology-based method [43]. PyMOL (3.0.4) visualization revealed significant carbon backbone shifts at mutation sites, particularly structural changes in V3593F and L3829F, with V3593F disrupting hydrogen bonding. This indicates that the mutations may have an effect on the structure and other properties of the proteins.

The structural and functional impacts of missense mutations were predicted using an integrated computational approach combining Missense3D, PolyPhen-2, SIFT, MuPro, SAAFEC, DUET, and PredyFlexy servers. These analyses evaluated protein stability, structural flexibility, and folding patterns. Molecular modeling revealed that the V3593F mutation completely disrupted hydrogen bonding networks, resulting in an 81.864 Å^3^ reduction in cavity volume that likely impedes substrate binding. Concurrently, the R3821K mutation shifted the residue from a buried to an exposed conformational state, potentially altering protein–protein interaction interfaces. Consensus predictions from Missense3D and DUET indicated that both mutations significantly decreased NSP6 structural stability, thereby impairing its biological function through combined steric hindrance and thermodynamic destabilization mechanisms. Elevated B-factor values at these sites in XBB.1.16, BA.2.86, and JN.1 indicated that the mutations may increase local flexibility and conformational instability [44]. Antigenicity analysis identified typical B-cell and CTL epitopes across all five variants of NSP6, with divergent epitope profiles potentially linked to pathogenicity differences, suggesting viral-infected cells may incur damage via immune or inflammatory responses.

Respiratory epithelial cells serve as the primary targets for viral infections in the airways [45]. Studies indicate that SARS-CoV-2 infection causes more severe damage to alveolar epithelial cells compared to other pulmonary cell types, with more direct and prolonged impacts on respiratory function [46].

Comparative transcriptomic analysis of A549 cells expressing WT-NSP6 versus variant NSP6 proteins (XBB.1.16, BA.2.86, JN.1) revealed distinct host signaling pathway modulations mediated by viral evolution: XBB.1.16-NSP6 induced the highest number of differential genes (517), with significant enrichment in vascular morphogenesis, glucocorticoid response, and PI3K-AKT pathways, suggesting its potential to exacerbate pathological damage through enhanced vascular permeability and suppression of host inflammatory responses; BA.2.86-NSP6 predominantly downregulated genes enriched in viral receptor activity and JAK-STAT signaling, suggesting that it may achieve immune escape by inhibiting the host immune recognition signaling pathway; JN.1-NSP6, although minimally different (only 12 down-regulated genes) compared to WT-NSP6, the differential genes were enriched in cancer transcriptional dysregulation and p53-mediated cellular senescence pathways, suggesting that it may establish persistent infection by regulating cellular senescence-related pathways.These findings highlight functional divergence among SARS-CoV-2 NSP6 variants through evolutionarily optimized regulation of host inflammatory, immune, and senescence signaling networks, providing novel insights into variant-specific pathogenic mechanisms.

A defining virological characteristic of SARS-CoV-2 involves strategic disruption of type I interferon (IFN)-dependent antiviral defense mechanisms [47]. IFN signaling serves as a critical modulator of cytokine storm pathogenesis, a hyperinflammatory cascade that is closely linked to the advancement of COVID-19 to acute respiratory distress syndrome (ARDS) and lethal consequences [48]. In A549 cells, transfection of the WT-NSP6 plasmid inhibited antiviral factor expression and suppressed p-IRF3/p-STAT1, aligning with prior findings [49]. Transfection of Alpha-NSP6, BA.2.86-NSP6, and JN.1-NSP6 plasmids similarly inhibited p-STAT1 without affecting IRF3 phosphorylation, consistent with Bills et al.’s findings that the NSP6 variant (ΔSGF) enhances suppression of host IFN signaling pathway activity [50]. Moreover, Alpha-NSP6 exhibits a stronger inhibitory effect on the type I interferon signaling pathway in cells, whereas XBB.1.16-NSP6 shows no impact on the cellular type I interferon signaling response, which may consequently influence the virus’s immune evasion capability.

SARS-CoV-2 infection leads to DNA damage and genomic instability during mammalian cell replication by disrupting normal cellular functions while impairing DNA repair mechanisms [51]. Experimental evidence demonstrates that SARS-CoV-2 suppresses the DNA damage response through interference with the recruitment of critical cell cycle regulators and DNA repair processes, ultimately compromising adaptive immunity [52]. The p53 signaling pathway emerges as one of the most important pathways during SARS-CoV-2 infection, with lower p53 levels correlating with severe respiratory pathologies, suggesting its protective role in pulmonary disorders [52]. Upon DNA damage, p53 regulates the expression of multiple genes to curb viral propagation [53]. However, viral infections, like SARS-CoV-2, can disturb cell cycle checkpoints and modify the activity of crucial proteins, including p53 [51]. Like other viruses, SARS-CoV-2 hijacks host metabolic pathways to synthesize replication-essential proteins, triggering aberrant activation of signaling cascades. Importantly, SARS-CoV-2 infection causes hyperactivation of the AKT-mTOR pathway in vitro, a process implicated in its disease mechanism [54].

In this study, transfection of cells with plasmids encoding nsp6 genes from various viral variants revealed differential regulation of p-AKT and p-mTOR. WT-NSP6 transfection in A549 cells elevated p-AKT and p-mTOR protein levels, whereas treatment with a p53 inhibitor abolished Alpha-NSP6- and BA.2.86-NSP6-induced p-mTOR elevation. Critically, JN.1-NSP6 failed to activate the AKT-mTOR pathway in A549 cells under all tested conditions. These findings indicate that p53 suppresses Alpha-NSP6- and BA.2.86-NSP6-mediated AKT-mTOR activation in A549 cells, suggesting variant-specific upstream regulatory mechanisms of NSP6. Viral infections may disrupt p53-mediated biochemical responses or signaling transduction, implying that mitigating such interference could constitute an effective antiviral strategy. Consequently, emphasizing p53′s therapeutic potential in coronavirus infections represents a significant opportunity to respond to the global health challenges posed by COVID-19 and emerging viral pathogens [55].

## 5. Conclusions

In summary, bioinformatics and cellular assays elucidated how NSP6 mutations alter protein structure and disrupt signaling pathways, revealing novel understandings of the pathogenic evolution of SARS-CoV-2 variants and informing strategies for pandemic control.

## Figures and Tables

**Table 1:** Online tools and URLs for bioinformatics analysis.

Online Tools	Functional Predictions	Web Address
TMHMM-2.0	Transmembrane spiral	https://services.healthtech.dtu.dk/service.php?TMHMM-2.0 (accessed on 22 August 2024)
Protter	protein topology	https://wlab.ethz.ch/protter/# (accessed on 22 August 2024)
SOPMA	Secondary structure [13,14]	https://npsa-prabi.ibcp.fr/cgi-bin/npsa_automat.pl?page=npsa_sopma.html (accessed on 26 August 2024)
PSIPRED		http://bioinf.cs.ucl.ac.uk/psipred/ (accessed on 27 August 2024)
I-TASSER	Three-tier structure [15,16,17]	https://zhanggroup.org/I-TASSER/ (accessed on 2 September 2024)
SAVES v6.0	Model quality assessment [18,19]	https://saves.mbi.ucla.edu/ (accessed on 2 September 2024)
SIFT	Protein function [20,21]	https://sift.bii.a-star.edu.sg/ (accessed on 3 September 2024)
Polyphen-2		http://genetics.bwh.harvard.edu/pph2/ (accessed on 3 September 2024)
Mupro	Protein stability [22,23,24,25,26,27]	https://mupro.proteomics.ics.uci.edu/ (accessed on 10 September 2024)
TM-Align		https://zhanggroup.org/TM-align/ (accessed on 10 September 2024)
DUET		https://biosig.lab.uq.edu.au/tools (accessed on 10 September2024)
SAAFEC		http://compbio.clemson.edu/SAAFEC-SEQ/ (accessed on 17 September 2024)
PredyFlexy	Protein flexibility [28]	https://www.dsimb.inserm.fr/dsimb_tools/predyflexy/ (accessed on 17 September 2024)
Immunomedicine Group	Epitope	http://imed.med.ucm.es/Tools/antigenic.pl (accessed on 10 October 2024)
IEDB	B-cell antigenic epitopes [29]	https://www.iedb.org/ (accessed on 10 October 2024)
CTL	T-cell antigenic epitopes [30]	https://nextgen-tools.iedb.org/tc1 (accessed on 10 October 2024)
SYFPEITHI		http://www.syfpeithi.de/bin/MHCServer.dll/EpitopePrediction.htm (accessed on 10 October 2024)

**Table 2:** Missense3D-predicted potentially deleterious amino acid variations.

Mutant Site	Relative to WT	Relative to Alpha
V3593F	Buried H-bond breakage	Cavity altered
R3821K	Buried/exposed switch	No structural damage detected
L3829F	No structural damage detected	No structural damage detected

**Table 3:** Polyphen-2 and SIFT predictions of structural and functional impacts of mutations.

Mutant Site	Relative to WT		Relative to Alpha		Relative to BA.2.86	
	Polyphen-2	SIFT	Polyphen-2	SIFT	Polyphen-2	SIFT
V3593F	0.181	0.03	0.181	0.06	-	-
R3821K	0.98	0.00	0.995	0	0.998	0.06
L3829F	0.988	0.19	0.988	0	-	-

Note: Polyphen-2 predicts the likelihood of amino acid substitutions altering protein structure/function (scores closer to 1.0 indicate higher probability). SIFT evaluates whether substitutions affect protein function (scores ≤ 0.05 suggest non-neutral, disruptive effects; lower scores indicate greater disruption).

**Table 4:** Mupro, DUET, TM-Align, and SAAFEC predictions of protein stability.

	Mutant Site	Relative to WT			Relative to Alpha			Relative to BA.2.86
Platforms		V3593F	R3821K	L3829F	V3593F	R3821K	L3829F	R3821K
Mupro (△△G)		−1.46	−1.31	−0.93	−1.46	−1.31	−0.93	−1.31
DUET (△△G)		−1.68	−1.665	−1.124	−1.518	−0.97	−1.199	−1.449
TM-Align (RMSD)		0.83	0.81	0.77	0.75	0.58	0.64	0.71
SAAFEC (△△G)		−0.39	−0.91	−0.76	−0.3	−0.82	−0.79	−0.82

Note: Mupro, DUET, and SAAFEC predict stability changes (ΔΔG < 0: reduced stability; ΔΔG > 0: increased stability). TM-Align assesses mutation impacts via RMSD (values > 0.15 imply potential functional effects).

**Table 5:** PredyFlexy-predicted B-factor values reflecting protein flexibility.

	Mutant Site	3593		3821		3829	
Variant		V	F	R	K	L	F
WT		−0.510		−0.248		0.081	
Alpha		−0.510		−0.248		0.081	
XBB.1.16		−0.510		−0.246			0.081
BA.2.86			−0.507	−0.248		0.081	
JN.1			−0.507		−0.248	0.081	

Note: The B-factor quantifies conformational flexibility in crystallographic models; higher values in-dictate greater flexibility and reduced stability in specific regions.

## Data Availability

Data are contained within the article. The data presented in this study are available on request from the corresponding author.

## Supplementary Materials

The following supporting information can be downloaded at https://www.mdpi.com/article/10.3390/cimb47050361/s1.
