# Supplementary material for: Impact of SARS-CoV-2 Variant NSP6 on Pathogenicity: Genetic Analysis and Cell Biology
Source: Curr Issues Mol Biol. 2025 May 14;47(5):361. doi: 10.3390/cimb47050361 (PMC12109688; doi:10.3390/cimb47050361)
Supplement: Supplementary file 1 [file cimb-47-00361-s001.zip › cimb-3587603-supplementary.pdf]

Supplementary Materials

Table S1. qRT-PCR primer sequences.

Gene		Primer Sequences（5'-3'）
pcDNA3.1	F	TATAAGCAGAGCTCTCTGGC
	R	GCAAACAACAGATGGCTGG
GAPDH	F	TCCAAAATCAAGTGGGGCGA
	R	AAATGAGCCCCAGCCTTCTC
ISG56	F	GAAGGATGGGCCTTGCTGAA
	R	GCGATAGGCAGAGATCGCAT
CXCL10	F	GCCATTCTGATTTGCTGCCT
	R	ATGCTGATGCAGGTACAGCG
ISG15	F	ATCACCCAGAAGATCGGCGT
	R	GGTTCGTCGCATTTGTCCAC
ISG54	F	GAGCGAAGGTGTGCTTTGAG
	R	GAGGGTCAATGGCGTTCTGA
IFN β	F	ACGCCGCATTGACCATCTAT
	R	GTCTCATTCCAGCCAGTGCT
IRF9	F	GCCCTACAAGGTGTATCAGTTG
	R	TGCTGTCGCTTTGATGGTACT
ISG20	F	CTCCTGAAAGGCAAGCTGGT
	R	TTGTGTAGCCGCTCATGTCC
IL8	F	CTCTTGGCAGCCTTCCTGATTTC
	R	GGGTGGAAAGGTTTGGAGTATGTC
IL6	F	CCCCTGACCCAACCACAAAT
	R	ATTTGCCGAAGAGCCCTCAG
HSPA6	F	CGGTTCTCTCCATTGACGCT
	R	CCGGTTGTCGAAGTCCTCTC
INHBA	F	CCTTGCTTTGGCTGAGAGGA
	R	GGTACATCCTTTGGGAGGGC
IL11	F	GAACTGTGTTTGCCGCCTG
	R	GAGGGTCTGGGGAAACTCG
4-Mar	F	TGTGATGGCTCGGTCAAGTG
	R	GTGCTTATGGCGATGACGTG
IL7R	F	CACGATGTAGCTTACCGCCA
	R	ATACATTGCTGCCGGTTGGA
HDAC9	F	TTCTGTGGCTGCTGTAGGAT
	R	TAAAGGTGAGATGGGCTCCAG
IGFBP1	F	CCTTTGGGACGCCATCAGTA
	R	GAGTTCTATTCGGCAGGGCT
AREG	F	CGCTCTTGATACTCGGCTCA
	R	CCCCAGAAAATGGTTCACGC
TC1	F	GGAGGTGCTCCACTTTCCAA
	R	TGGAGAAGTGATTGGGCAGC
CD74	F	CCCATGCAGAATGCCACCAA

	R	GGGTCCTCCAGTTCCAGTGA
HOGA1	F	GAAGGTGGACATTGCGGGTA
	R	AGCTCCTCGGAAGGGGAAG
TMPRSS5	F	ATGCATGCCAGGGAGATAGC
	R	CAGCTACCTTGGCGTAGACA
SELENOP	F	AAAGCTCCTTATGTAAGCAACCC
	R	ACAGTCACTGAACCATTGGAGT
HSPA6	F	CCAGAGGAACGCCACTATCC
	R	CACCTGGATGAAGACCCCAG
INHBA	F	TGGAGTGTGATGGCAAGGTC
	R	CCAGTCATTCCAGCCGATGT
IL11	F	GAACTGTGTTTGCCGCCTG
	R	GAGGGTCTGGGGAAACTCG
EGR3	F	GACTCCCCTTCCAACTGGTG
	R	ATACATGGCCTCCACGTCAC
RASD1	F	CCTACACGCCTACCATCGAG
	R	GGTGTCGAGGATCTGTGAGG
PZP	F	AGGAGACTTGAAGCCAGCAA
	R	GGGCGATATACTCAGCAACCA
VIT	F	AAGCCTATCAGAGGCCACCT
	R	GGGGATGCTGGTGGTAGAAG
PLIN4	F	CCAGTTCCAGGACCACAGAC
	R	CTACACTGAGCACATCCGGG
CACNB2	F	GCGAGTACGGGTGTCCTATG
	R	CCGGTCCTCCTCCAGAGATA
IGFBP3	F	AGAAAAAGCAGTGTCGCCCT
	R	TCCTTCCCCTTGGTGGTGTA
NDRG1	F	CTCTCCTCAAGATGGCGGAC
	R	ATGTATCCCATGCCCTGCAC
FIBCD1	F	TGAGAATGGCACGGCCTATG
	R	CTGTGCTTCAGGAGGGAGTC
TMEM158	F	ACCGTAAGCCCATTGAGTCC
	R	CAGGAAGCCGGCGATGAT
EGLN	F	CCTGTCTGCACGAGGCAAT
	R	ACCATCACCGTTGGGGTTG
NEURL1BJ6	F	GGCCAAAGGCAAGAACGTG
	R	GAACGTGACGCCATTGCAG
UCN2	F	CGCGCATTGTCCTATCGCT
	R	CGGGCTTGCTCCAGTAAGA
RIMKLA	F	ACCTCGGCCTCCAGCTAAA
	R	GTGTCGCAGGACAGTGATGT

Table S2. Summary of WHO on dominant variants of VOI, VUM.

Date	VOI	VUM
2023.03.22	XBB.1.5	BQ.1/BA.2.75/CH.1.1/XBB/XBF
2023.03.30	XBB.1.5	BQ.1/BA.2.75/CH.1.1/XBB/XBF/XBB.1.16
2023.04.06	XBB.1.5	BQ.1/BA.2.75/CH.1.1/XBB/XBF/XBB.1.16/XBB.1.9.1
2023.04.13	XBB.1.5	BA.2.75/CH.1.1/BQ.1/XBB/XBB.1.16/XBB.1.9.1/XBF
2023.04.20	XBB.1.5/XBB.1.16	BA.2.75/CH.1.1/BQ.1/XBB/XBB.1.9.1/XBF
2023.04.27	XBB.1.5/XBB.1.16	BA.2.75/CH.1.1/BQ.1/XBB/XBB.1.9.1/XBF/XBB.1.9.2
2023.05.04	XBB.1.5/XBB.1.16	BA.2.75/CH.1.1/BQ.1/XBB/XBB.1.9.1/XBF/XBB.1.9.2
2023.05.11	XBB.1.5/XBB.1.16	BA.2.75/CH.1.1/BQ.1/XBB/XBB.1.9.1/XBB.1.9.2
2023.05.18	XBB.1.5/XBB.1.16	BA.2.75/CH.1.1/BQ.1/XBB/XBB.1.9.1/XBB.1.9.2/XBB.2.3
2023.05.25	XBB.1.5/XBB.1.16	BA.2.75/CH.1.1/BQ.1/XBB/XBB.1.9.1/XBB.1.9.2/XBB.2.3
2023.06.01	XBB.1.5/XBB.1.16	BA.2.75/CH.1.1/BQ.1/XBB/XBB.1.9.1/XBB.1.9.2/XBB.2.3
2023.06.08	XBB.1.5/XBB.1.16	BA.2.75/CH.1.1/BQ.1/XBB/XBB.1.9.1/XBB.1.9.2/XBB.2.3
2023.06.15	XBB.1.5/XBB.1.16	BA.2.75/CH.1.1/BQ.1/XBB/XBB.1.9.1/XBB.1.9.2/XBB.2.3
2023.06.22	XBB.1.5/XBB.1.16	BA.2.75/CH.1.1/XBB/XBB.1.9.1/XBB.1.9.2/XBB.2.3
2023.06.29	XBB.1.5/XBB.1.16	BA.2.75/CH.1.1/XBB/XBB.1.9.1/XBB.1.9.2/XBB.2.3
2023.07.06	XBB.1.5/XBB.1.16	BA.2.75/CH.1.1/XBB/XBB.1.9.1/XBB.1.9.2/XBB.2.3
2023.07.13	XBB.1.5/XBB.1.16	BA.2.75/CH.1.1/XBB/XBB.1.9.1/XBB.1.9.2/XBB.2.3
2023.07.20	XBB.1.5/XBB.1.16	BA.2.75/CH.1.1/XBB/XBB.1.9.1/XBB.1.9.2/XBB.2.3/EG.5
2023.07.27	XBB.1.5/XBB.1.16	BA.2.75/CH.1.1/XBB/XBB.1.9.1/XBB.1.9.2/XBB.2.3/EG.5
2023.08.03	XBB.1.5/XBB.1.16	BA.2.75/CH.1.1/XBB/XBB.1.9.1/XBB.1.9.2/XBB.2.3/EG.5
2023.08.10	XBB.1.5/XBB.1.16/EG.5	BA.2.75/CH.1.1/XBB/XBB.1.9.1/XBB.1.9.2/XBB.2.3
2023.08.17	XBB.1.5/XBB.1.16/EG.5	BA.2.75/CH.1.1/XBB/XBB.1.9.1/XBB.1.9.2/XBB.2.3/BA.2.86
2023.08.25	XBB.1.5/XBB.1.16/EG.5	BA.2.75/CH.1.1/XBB/XBB.1.9.1/XBB.1.9.2/XBB.2.3/BA.2.86
2023.09.29	XBB.1.5/XBB.1.16/EG.5	BA.2.75/CH.1.1/XBB/XBB.1.9.1/XBB.1.9.2/XBB.2.3/BA.2.86
2023.10.27	XBB.1.5/XBB.1.16/EG.5	XBB/XBB.1.9.1/XBB.1.9.2/XBB.2.3/BA.2.86/DV.7
2023.11.24	XBB.1.5/XBB.1.16/EG.5/BA.2.86	XBB/XBB.1.9.1/XBB.1.9.2/XBB.2.3/DV.7
2023.12.22	XBB.1.5/XBB.1.16/EG.5/BA.2.86/JN.1	XBB/XBB.1.9.1/XBB.1.9.2/XBB.2.3/DV.7
2024.01.19	XBB.1.5/XBB.1.16/EG.5/BA.2.86/JN.1	XBB/XBB.1.9.1/XBB.1.9.2/XBB.2.3/DV.7
2024.02.16	XBB.1.5/XBB.1.16/EG.5/BA.2.86/JN.1	XBB/XBB.1.9.1/XBB.2.3
2024.03.15	XBB.1.5/XBB.1.16/EG.5/BA.2.86/JN.1	XBB/ XBB.1.9.1/XBB.2.3
2024.04.12	XBB.1.5/XBB.1.16/EG.5/BA.2.86/JN.1	——
2024.05.17	XBB.1.5/XBB.1.16/EG.5/BA.2.86/JN.1	JN.1.7/ JN.1.18/ KP.2/ KP.3
2024.06.17	EG.5/BA.2.86/JN.1	JN.1.7/ JN.1.18/ KP.2/ KP.3
2024.07.15	BA.2.86/JN.1	JN.1.7/JN.1.18/KP.2/KP.3/LB.1
2024.08.17	BA.2.86/JN.1	JN.1.7/JN.1.18/KP.2/KP.3/ KP.3.1.1/ LB.1
2024.09.17	BA.2.86/JN.1	JN.1.7/JN.1.18/KP.2/KP.3/ KP.3.1.1/ LB.1
2024.10.09	BA.2.86/JN.1	JN.1.7/JN.1.18/KP.2/KP.3/KP.3.1.1/LB.1/XEC
2024.11.06	BA.2.86/JN.1	JN.1.7/JN.1.18/KP.2/KP.3/ KP.3.1.1/LB.1/XEC
2024.12.24	JN.1	JN.1.18/KP.2/KP.3/KP.3.1.1/LB.1/XEC

Table S3. Proportion of secondary structure of NSP6 protein of variants.

Variant	α-helices	Extended chains	β-folding	Random coils
WT	50.69%	26.21%	5.17%	17.93%
Alpha	52.61%	24.74%	5.92%	16.72%
JN.1	54.01%	25.09%	5.92%	14.98%
BA.2.86	54.70%	23.69%	4.88%	16.72%
XBB.1.16	54.70%	20.21%	5.57%	19.51%

Table S4. Predicted T-cell antigenic epitope scores and factor sequences of NSP6 proteins.

Alleles	Sequences	Start	WT		Alpha		JN.1		BA.2.86		XBB.1.16	
			Score[1]	Score[2]	Score[1]	Score[2]	Score[1]	Score[2]	Score[1]	Score[2]	Score[1]	Score[2]
HLA-A*02:01	FLLPSLATV	70	0.99	33	0.99	33	0.99	33	0.99	33	0.99	33
HLA-A*02:01	TLMNVLTLV	138			0.92	25	0.92	25	0.92	25	0.92	25
HLA-A*02:01	TLMNVLTLV	141	0.92	25								
HLA-A*02:01	SMWALIISV	160			0.88	28	0.88	28	0.88	28	0.88	28
HLA-A*02:01	SMWALIISV	163	0.88	28								
HLA-A*02:01	FLARGIVFM	181			0.85	25	0.85	25	0.85	25	0.85	25
HLA-A*02:01	FLARGIVFM	184	0.85	25								
HLA-A*02:01	ILTSLLVLV	18	0.80	28	0.80	28	0.73	26	0.73	26	0.80	28
HLA-A*02:01	LLLTILTSL	14	0.79	30	0.79	30	0.79	30	0.79	30	0.79	30
HLA-A*02:01	KLNIKLLGV	267			0.69	29	0.69	29	0.69	29	0.69	29
HLA-A*02:01	KLNIKLLGV	270	0.68	29								
HLA-A*02:01	GLLPPKNSI	255			0.56	27	0.56	27	0.56	27	0.66	26
HLA-A*02:01	GLLPPKNSI	258	0.56	27								

1: IEDB-T Cell Prediction predicts T-cell antigenic epitope scores, selecting antigenic epitopes > 0.5;

2: SYFPEITHI predicts T-cell antigenic epitope scores, selecting antigenic epitopes > 25.

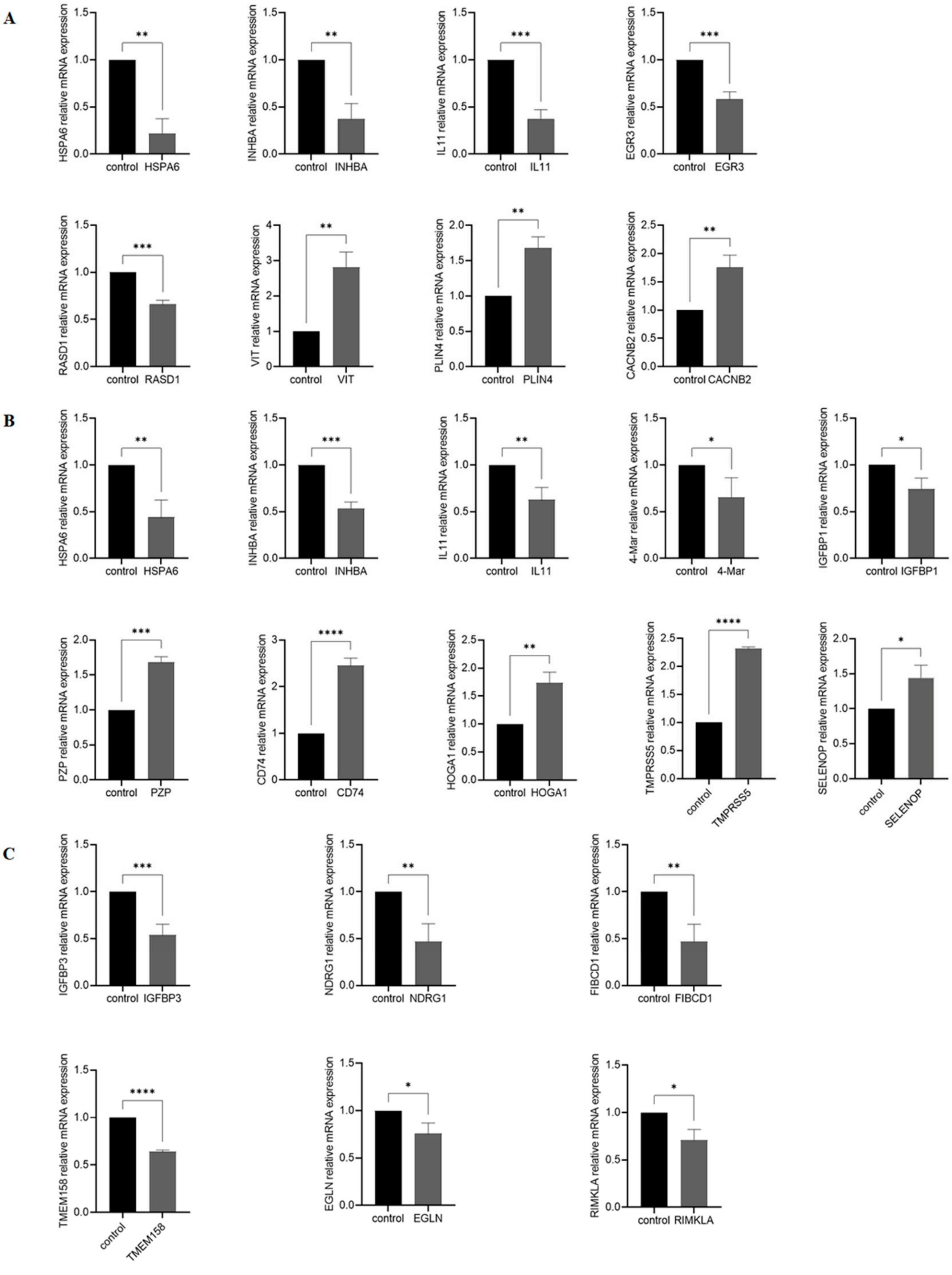

Figure S1. Validation of transcriptomic differentially expressed genes. (**A**-**C**) validation of differentially expressed genes in the XBB.1.16-NSP6, BA.2.86-NSP6, and JN.1-NSP6 groups compared to the WT-NSP6 control, respectively. Statistical significance: * $p < 0.05$, ** $p < 0.01$, *** $p < 0.005$, **** $p < 0.001$.
